
JOURNAL INFORMATION
==============================
NLM Title Abbreviation: Am J Trop Med Hyg
Journal ID: Am J Trop Med Hyg
NLM Journal ID: 0370507
Journal ID: tpmd

The American Journal of Tropical Medicine and Hygiene

ISSN: 0002-9637
EISSN: 1476-1645
Publisher: The American Society of Tropical Medicine and Hygiene

ARTICLE INFORMATION
==============================
PMCID: PMC10919234
DOI: 10.4269/ajtmh.1103stoc
Article ID: tpmd1103stoc
Article version: 1
Subjects: Other

Contents

Print publication date: 2024 Mar
Electronic publication date: 2024 Mar 5
Volume: 110
Issue: 3 Suppl
Issue title: Improving the Quality of Malaria Service Delivery: Lessons Learned from the U.S. President’s Malaria Initiative Impact Malaria Project
First page: ii
Last page: ii
Copyright: © The American Society of Tropical Medicine and Hygiene
Copyright year: 2024
License: This is an open-access article distributed under the terms of the Creative Commons Attribution (CC-BY) License, which permits unrestricted use, distribution, and reproduction in any medium, provided the original author and source are credited.
License URL: https://creativecommons.org/licenses/by/4.0/

Page count: 1

==============================

### Improving the Quality of Malaria Service Delivery: Lessons Learned from the U.S. President's Malaria Initiative Impact Malaria Project

**Guest Editor: S. Patrick Kachur**

### CONTENTS

**The U.S. President's Malaria Initiative's Support for Improving the Quality of Malaria Case Management Services: Fifteen Years of Progress and Learning**
Lawrence M. Barat, Nicole Whitehurst, Meera Venkatesan, Kim Connolly, Emmanuel Yamo, Paul Psychas, and Yves-Marie Bernard **1**

**Can Outreach Training and Supportive Supervision Improve Competency in Malaria Service Delivery? An Evaluation in Cameroon, Ghana, Niger, and Zambia**
Ruth A. Ashton, Matt Worges, Albert Zeh Meka, Paul Yikpotey, Irenee Domkam Kammogne, Pascalina Chanda-Kapata, Caroline Vanderick, Elizabeth Streat, and Joshua Yukich **10**

**Outreach Training and Supportive Supervision for Quality Malaria Service Delivery: A Qualitative Evaluation in 11 Sub-Saharan African Countries**
Robin Altaras, Matt Worges, Sabrina La Torre, Bala M. Audu, Grace Mwangi, Albert Zeh-Meka, Paul Yikpotey, Irenee Domkam Kammogne, Pascalina Chanda-Kapata, Caroline Vanderick, Joshua Yukich, and Elizabeth Streat **20**

**Clinical Outreach Training and Supportive Supervision Quality-of-Care Analysis: Impact of Readiness Factors on Health Worker Competencies in Malaria Case Management in Cameroon, Mali, and Niger**
Yves-Marie Bernard, Jehan Ahmed, Jadmin Mostel, Thierno Ba, Annie Coriolan Ciceron, Moses Busiga, Aissata Koné, Beh Kamaté, Fatoumata Sidibé, Chebou Diallo, Alzouma Makayi, Daniel Christian Koko, Arouna Djibrilla, Joël Ateba, Eric Tchinda Meli, Christophe Tchadjeu, Kevin Griffith, Jordan Burns, and Lawrence M. Barat **35**

**Use of Supervision Data to Improve Quality of Care for Malaria in Pregnancy: Experience in Six African Countries**
Katherine Wolf, Jadmin Mostel, Lolade Oseni, Patricia Gomez, Tabitha Kibuka, Courtney Emerson, Julie R. Gutman, Ashley Malpass, Susan Youll, Jean Yves Mukamba, Eric Tchinda Meli, Dorothy Achu, Paul Tjek, Jean Louis Assa, Mamadou Silue, Méa Antoine Tanoh, Colette Kokrasset-Yah, Felicia Babanawo, Amos Asiedu, Mildred Komey, Paul Boateng, Maureen Mabiria, Augustine Ngindu, Peter Njiru, Ahmeddin Hassan Omar, Fatoumata A. Sidibe, Chebou Diallo, Beh Kamate, Aissata Kone, Sanoussi Elisha, Arouna Djibrilla Maiga, Alzouma Ibrahim Mayaki, Fati Tidjani Issa Gana, and Gladys Tetteh **42**

**How Outreach Training and Supportive Supervision (OTSS) Affect Health Facility Readiness and Health-Care Worker Competency to Prevent and Treat Malaria in Niger: A Secondary Analysis of OTSS Data**
Daniel Koko, Djibrilla Arouna, Yves-Marie Bernard, Thierno Ba, Jadmin Mostel, Yahaya Abdou, Eric Coulibaly, Zilahatou Bahari-Tohon, and Lawrence M. Barat **50**

**Understanding Antenatal Care Service Quality for Malaria in Pregnancy through Supportive Supervision Data in Tanzania**
Goodluck Elias Tesha, Stella Makwaruzi, Rachel Haws, Jadmin Mostel, Abdallah Lusasi, Samwel Lazaro, Sijenunu Mwaikambo, Frank Chacky, Erik Reaves, Chonge Kitojo, Naomi Serbantez, Gladys Tetteh, Katherine Wolf, and Lolade Oseni **56**

**Experiences in Improving the Quality of Community-Based Fever Management from Three Malaria-Endemic African Countries**
Jehan Ahmed, Adam Nothem, Jadmin Mostel, Annie Ciceron, Luigi Nuñez, Oméga Raobela, Andry Patrick Raoiliarison, Sosten Lankhulani, John Munthali, Mady Cissoko, Beh Kamaté, Oumar Yattara, Samba Coumaré, and Katherine Wolf **66**

**Impact of the Severe Malaria "Champions Program" on the Management of Severe Malaria Cases in 12 Hospitals of the North and Far North Regions of Cameroon**
Eric Tchinda Meli, Yves-Marie Bernard, Joël Marcellin Ateba, Landry Tchoutang, Abas Mouliom, Annie Coriolan Ciceron, Jadmin Mostel, Christophe Tchadjeu, Olivier Palata, Mohamadu Wirngo, Aissata Fofana, and Lawrence M. Barat **76**

**EDITORIAL**

**Outreach Training and Supportive Supervision: A Package of Strategies That Improves the Quality of Malaria Services and Provides a Model for Monitoring and Evaluating Their Effective Implementation**
MaryKelly S. McCarthy and S. Patrick Kachur **83**

For more information about
the American Society of Tropical Medicine and Hygiene
please visit us at www.astmh.org

The American Journal of Tropical Medicine and Hygiene is
online at www.ajtmh.org
